# Feasibility and first-year findings of a pilot program of prostate cancer screening in Croatia (CROState)

**DOI:** 10.3325/cmj.2025.66.382

**Published:** 2025-12

**Authors:** Željko Kaštelan, Tomislav Kuliš, Igor Grubišić, Ivana Brkić Biloš, Maja Prutki, Marija Bubaš, Krunoslav Capak

**Affiliations:** 1 Department of Urology, University Hospital Center Zagreb, Zagreb, Croatia; 2 University of Zagreb School of Medicine, Zagreb, Croatia; 3 Department of Urology, Sestre Milosrdnice University Hospital Center, Zagreb, Croatia; 4 Croatian Institute of Public Health, Zagreb, Croatia; 5 Clinical Department of Diagnostic and Interventional Radiology, University Hospital Centre Zagreb, Zagreb, Croatia; 6 Ministry of Health, Zagreb, Croatia

## Abstract

**Aim:**

To evaluate the feasibility, participation rate, diagnostic pathway performance, and early detection outcomes of the first year of the Croatian pilot prostate cancer screening program (CROState) implemented in Zagreb.

**Methods:**

This prospective pilot program invited men aged 55-69 years without a prior prostate cancer diagnosis or prostate-specific antigen (PSA) testing in the past 12 months. Recruitment was conducted by general practitioners. Men with PSA>4 ng/mL underwent repeat testing, and if PSA was elevated again, they were referred to one of two university hospitals for further evaluation. The diagnostic pathway included multiparametric magnetic resonance imaging, urological examination, and transperineal fusion biopsy when indicated. All confirmed cancer cases were reviewed by a multidisciplinary team.

**Results:**

A total of 5251 men were invited to the program, of whom 4930 (93.9%) participated in PSA testing. Elevated PSA was detected in 419 (8.5%). Only 157 (37.5%) men completed repeat PSA testing, and 123 men were referred for hospital evaluation. Eighty-eight patients completed advanced diagnostics, with 83 undergoing magnetic resonance imaging. Forty-two men proceeded to biopsy, of whom 27 had positive results (64.3%). Most cancers were clinically significant; only two men fulfilled criteria for active surveillance. The main challenge was incomplete adherence to repeat PSA testing.

**Conclusion:**

The CROState pilot demonstrated high initial participation and high detection rate of prostate cancer, with a few clinically insignificant tumors, when combining PSA testing with advanced imaging and targeted biopsy. Limited compliance with repeat PSA testing must be addressed before wider national implementation.

Since 2016, prostate cancer has been the most common malignant disease among Croatian men and the third leading cause of death (1,2). It is also the most prevalent cancer in the Western and developed world, which makes it a major public health concern (3).

The Europe’s Beating Cancer Plan by the European Council in December 2022 recommended establishing screening programs for prostate cancer in member countries (4). The EU-funded PRAISE-U project is implementing five different pilot programs to collect relevant data on how to effectively design screening programs (5). Croatia has recognized prostate cancer as an important issue, and the Ministry of Health established a working group to develop recommendations for a national screening program.

Since the introduction of prostate-specific antigen (PSA) testing in clinical practice, screening programs have undergone extensive research. Substantial experience has been gained through well-known population screening programs, such as ERSPC in Europe and PLCO in the USA (6,7). Early results were followed by disappointment due to overdiagnosis and overtreatment. However, after the 23-year follow-up, the ERSCP study has shown 13% lower prostate cancer mortality in the screening group (8). Moreover, significant progress has been made in addressing overtreatment. Robust evidence now supports active surveillance as a treatment option for low-risk cancers (9,10). Additionally, to minimize overdiagnosis, we currently have tools such as nomograms, multiparametric MRI, transrectal micro-ultrasound, and additional genetic tests.

Moreover, 16-year follow-up data from ERSPC and 21-year follow-up data from a Dutch cohort showed that 18 and 14 men, respectively, needed to be diagnosed to prevent one prostate cancer death (7,11). These figures are even more compelling than those for breast cancer screening (7,12).

Current guidelines by the European Association of Urology (EAU) recommend that a risk-adapted strategy for early detection is offered to a well-informed man with a life expectancy of at least 15 years (13). In a similar approach, the American Urological Association recommends PSA testing through shared decision-making with men aged 50-69 years (14).

The primary goal of screening programs is to reduce the rate of metastatic disease and mortality. A delayed diagnosis may lead to increased mortality and more complex treatment. Considering that the incidence of prostate cancer peaks in the 50s, while the mortality rate peaks in the 70s, the optimal target group for screening are men aged 50 to 60. The aim of this study is to evaluate the feasibility of a pilot program in the City of Zagreb, Croatia. Feasibility was measured through participation, repeat PSA compliance, and diagnostic pathway completion, as well as early prostate cancer detection.

## Patients and methods

Our working group was formed in May 2023. The team included representatives from all key stakeholders: urologists, oncologists, general practitioners (GPs), radiologists, nuclear medicine specialists, pathologists, medical biochemists, policy representatives, and IT specialists.

A smaller pilot program was implemented to identify and address potential challenges before expanding the program nationwide. The designed program aimed to provide the following benefits to the health care system:

• improve access to health care;

• improve access to modern diagnostic methods (all patients with elevated PSA should undergo multiparametric MRI and transperineal fusion biopsy);

• improve access to treatment options (offer treatment at experienced centers with robotic surgery and radiotherapy);

• ensure that all patients receive treatment decisions through a multidisciplinary team (MDT) consultation;

• reduce the rate of metastatic disease;

• reduce mortality rates.

### Pilot program overview

Participants were recruited through GP consultations. We initially planned to include 10 000 men for PSA testing. If PSA on the first test was elevated (>4 ng/mL), a second test was conducted within 3-5 weeks. This period was chosen to allow for normalization of transient PSA elevations related to inflammation or recent ejaculation while minimizing the risk of patient dropout. If the second test was also positive, the man was referred to the hospital system.

In the hospital system, patients were guided by a dedicated managing nurse through the following steps:

1. undergoing multiparametric MRI (unless medically contraindicated);

2. visiting a urologist;

3. undergoing a prostate biopsy if indicated;

4. undergoing a second urological consultation;

5. if diagnosed with prostate cancer, undergoing an MDT consultation for treatment recommendations.

Initially, 246 GP teams were enrolled following consultations with their respective health centers. However, after ten months, an additional 189 GP teams were included to meet the target number of screened patients.

The program overview was approved by the Ministry of Health and published only by the Croatian Institute of Public Health (15). The program received funding and official approval on March 1, 2024, and was launched on March 15, 2024, after resolving multiple IT issues.

The program included men aged 55-69 who had no prior prostate cancer diagnosis and had not undergone PSA testing in the past 12 months. If a man had previously elevated PSA levels outside the program and was already planned for urological consultation, he could also be included in the program after confirming a second elevated PSA test. This approach ensured better access to quality diagnostics and treatment planning.

**Figure 1:**
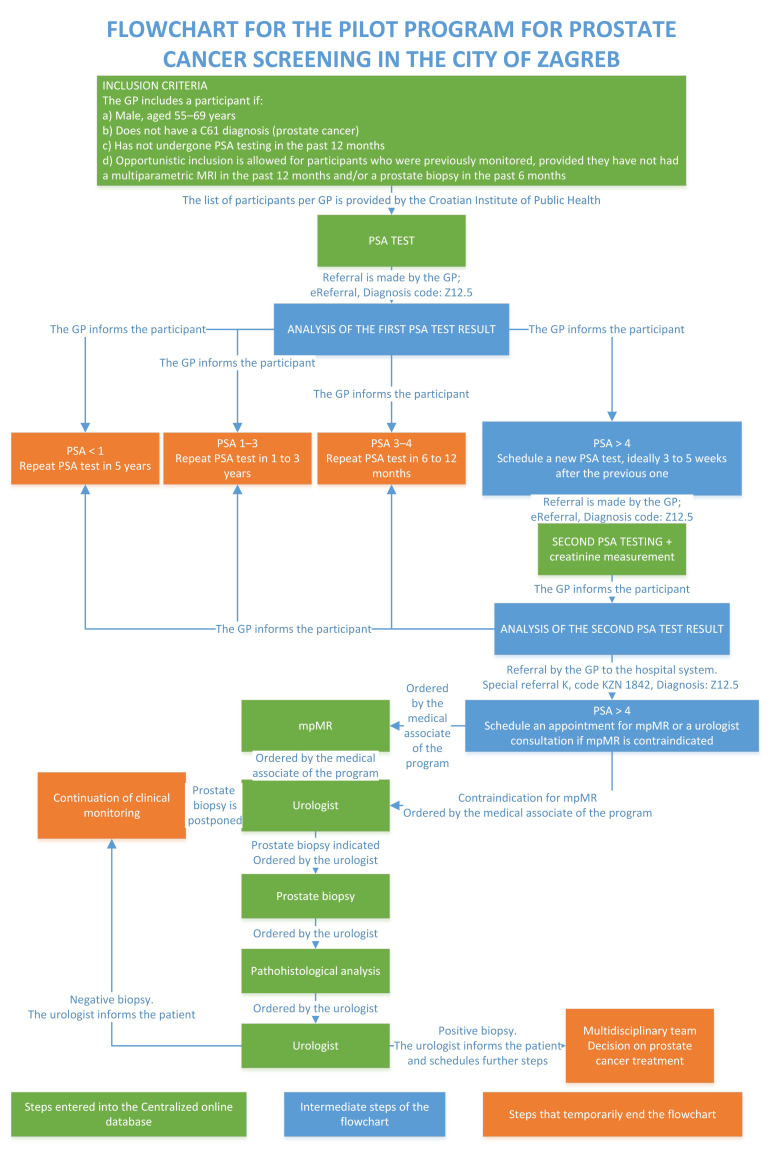
Flowchart for the pilot program for prostate cancer screening in the City of Zagreb. PSA – prostate-specific antigen; GP – general practitioner; MRI – magnetic resonance imaging; mpMR – multiparametric magnetic resonance.

According to the proposed program (Figure 1), men who did not have elevated PSA continued follow-up with their GPs based on their PSA level. If PSA was less than 1 ng/mL, repeat testing was recommended in 5 years; if PSA was 1-3 ng/mL, repeat testing was advised in 1-3 years; and if PSA was 3-4 ng/mL, repeat testing was recommended in 6-12 months.

Men with two elevated PSA values (n = 88) were referred to one of two university hospital centers in Zagreb. These hospitals have extensive experience in prostate cancer diagnosis and treatment. In 76 men, we performed 3-Tesla MRI, and in 9 men 1.5-Tesla MRI. All multiparametric MRI scans were performed using standard prostate imaging protocol (T2W, DWI, ADC maps, DCE sequences), and the PIRADS v. 2.1 scoring system was used for reporting (16).

Prostate biopsy was indicated in case of a positive digital rectal examination, and/or a PIRADS 4 or 5 lesion, and/or PSA density >0.15 ng/mL^2^. All prostate biopsies were performed using the transperineal approach, combining systematic and fusion-targeted cores. When multiparametric MRI was unavailable, additional cores were taken if suspicious areas were detected by micro-ultrasound during the procedure. The biopsy protocol was to take 2-3 targeted cores per lesion on MRI followed by 12 systematic cores. Both participating hospitals are equipped with a fusion system that has micro-ultrasound capability, and this feature was used when multiparametric MRI was not available. The diagnostic yield of this approach will be analyzed once a larger cohort of such patients becomes available.

If a prostate biopsy was not indicated, men were further followed by a urologist with the frequency of visits and repeat PSA measurements determined at the discretion of the treating urologist. Both hospitals have experienced MDTs that meet weekly to determine the best treatment for prostate cancer patients, including those in the pilot program.

This study is based on the analysis of anonymized data collected within the framework of the pilot prostate cancer screening program, officially approved and supported by the Ministry of Health of the Republic of Croatia. As this quality-assurance study is aimed at evaluating and improving an established public health program, separate ethical approval was not required for this analysis. No identifiable patient data were used.

### IT infrastructure

To minimize costs and avoid the need for clinicians to adapt to new platforms, computer systems existing in all participating health centers and hospitals were used. Fortunately, most GPs and both hospitals use the same hospital information system. Additionally, all medical documents must be uploaded to the centralized national health care database, which ensured streamlined data management.

The Croatian Institute of Public Health coordinated and monitored the program, conducting bimonthly data analyses. The main disadvantage of using multiple computer platforms for screening was the need to manually collect and analyze data. However, the major advantage was higher clinician participation, as they did not need to adapt to new IT systems.

## Results

During the first two months, GPs enrolled more than 3000 patients. By summer, 4500 patients had been included, but recruitment stagnated, which was initially attributed to summer vacations. However, further analysis in September suggested that the initial 246 GP teams had exhausted their eligible patient pool, partly due to regular PSA testing conducted through employer-provided checkups. By January 2025, an additional 189 GP teams were included, revitalizing the program.

### PSA test results

**Figure 2:**
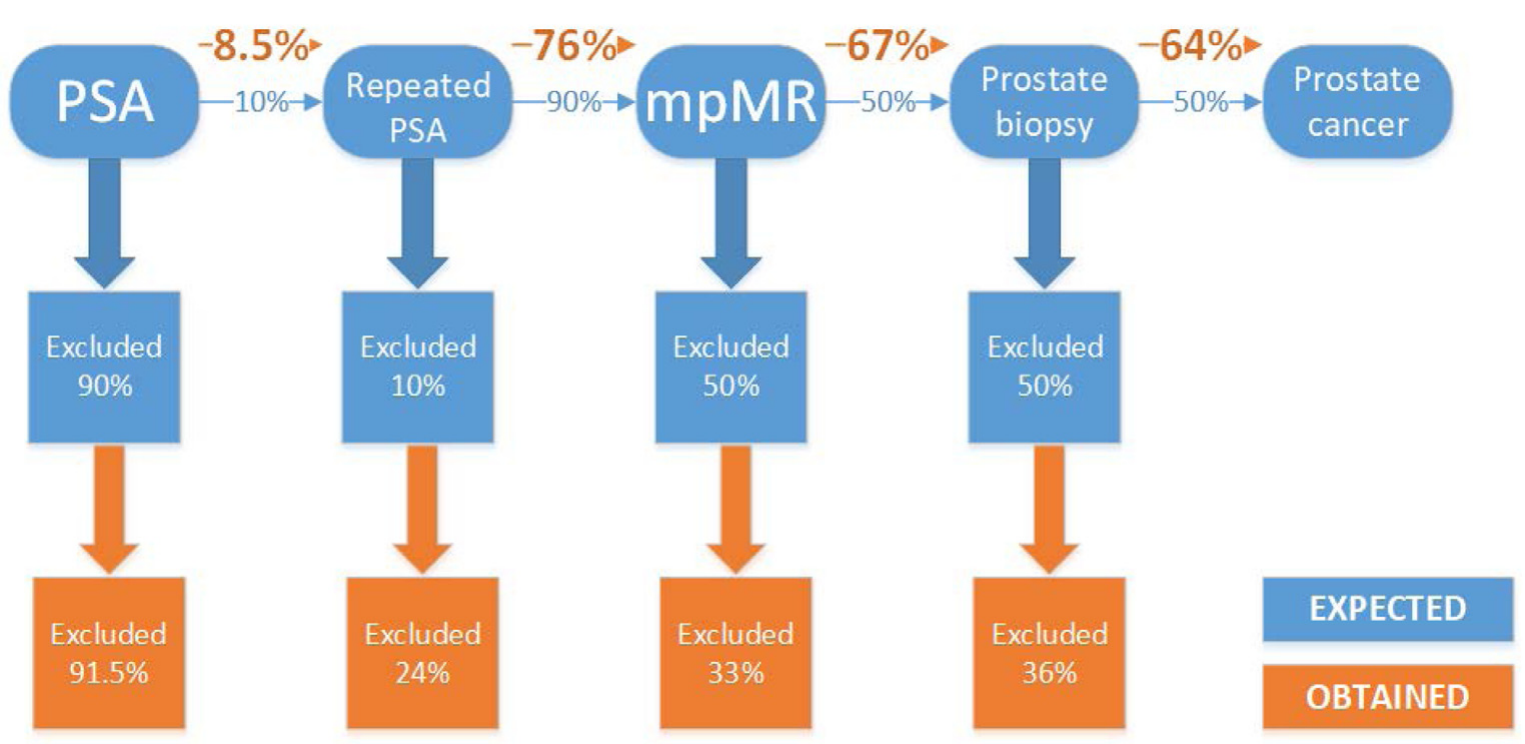
Expected vs obtained results in different steps of the program. Expected numbers are based on published data and clinical experience. mpMR – multiparametric magnetic resonance.

Of the 5251 invited men, 4930 (93.9%) underwent PSA testing. PSA was >4 ng/mL in 419 (8.5%) men. Of these, only 157 (37.5%) completed the required second PSA test, and 76% of them had persistently elevated PSA levels. A total of 123 men with elevated PSA results were referred for additional diagnostic evaluation in two participating hospitals. Of these, 120 men had two consecutive elevated PSA values obtained within the pilot program, while 3 men had an elevated PSA prior to program entry and underwent their second confirmatory test within the program. The number of positive repeat PSA tests was lower than expected based on published data and clinical experience (76% vs 90%), while the positivity rate for multiparametric MRI (67 vs 50%) and positivity rate for prostate biopsies (64% vs 50%) were higher (Figure 2).

### Multiparametric MRI and prostate biopsy results

Of 123 referred men at the time of this analysis, 88 completed the hospital diagnostic pathway (79 in Zagreb University Hospital Center and 9 in Sestre Milosrdnice University Hospital Center). Of these, 85 underwent multiparametric MRI (three did not, due to contraindications). PIRADS 3 was reported in 18 (21.2%), PIRADS 4 in 19 (22.4%), and PIRADS 5 in 20 (23.5%) patients. Overall, suspicious multiparametric MRI with PIRADS≥3 was reported in 67.1% of patients.

All 88 patients underwent urological evaluations, and 42 of them were referred for biopsy. All biopsies included fusion-targeted and systematic transperineal biopsy, with two micro-ultrasound-guided biopsies for those ineligible for multiparametric MRI. There were no biopsy-related complications, except transient hematuria and/or hematospermia.

A total of 27 biopsies were positive (64.3%). Pathology results and MDT recommendations are detailed in Table 1. Treatment outcomes are outside the scope of this paper.

**Table 1:** Pathology results of prostate biopsies and decisions for further treatment by a multidisciplinary team (MDT)

Gleason grade group	Number (%)
1	7 (25.9)
2	11 (40.8)
3	5 (18.5)
4	0 (0)
5	4 (14.8)
MDT recommendation	
Active surveillance*	2 (7.4)
Radical prostatectomy	19 (70.4)
Radiotherapy	4 (14.8)
Systemic treatment	2 (7.4)

*Decision according to the PRIAS protocol (19).

## Discussion

The implementation of the pilot prostate cancer screening program in Zagreb demonstrated both the feasibility and the challenges of such an initiative. The high participation rate (93.9%) suggests strong public interest and acceptance. However, the low rate of second PSA testing (37.5%) highlights a key area for improvement. Future efforts should focus on patient education and follow-up strategies to ensure compliance with testing protocols.

This pilot also revealed several logistical and organizational challenges. A considerable proportion of men did not proceed with the recommended second PSA testing, which may have limited the overall effectiveness of the screening process. Further investigation is needed to explain this low follow-up rate. Some participants might have sought further testing through standard health care channels, outside the pilot program, which led to incomplete data capture and reduced continuity of care.

Inviting men into the program through general practitioners proved to be a highly effective approach, especially when compared with other Croatian screening programs, which rely on invitation letters (17). However, there remains room for improvement. Strengthening communication with both GPs and the general public is essential to increase participation rates. Despite the initial momentum, we did not reach the planned number of 10 000 invitations within the first year. The pace of invitations fluctuated, with the highest inclusion rates observed during the initial months of the program.

The inclusion of multiparametric MRI and fusion biopsy helped mitigate concerns regarding overdiagnosis and overtreatment, ensuring a more targeted approach to identifying clinically significant prostate cancers (18). The referral of 88 patients to hospital care, with 42 undergoing biopsy, indicated that the screening process effectively identified at-risk individuals. Notably, 64.3% of biopsies were positive, demonstrating that the diagnostic approach of two PSA tests followed by multiparametric MRI provides optimal biopsy indication. Urologists should follow EAU guidelines when deciding on prostate biopsy (13). Only two patients met the clinical and pathological criteria for active surveillance according to the PRIAS protocol (19), which further supports the notion that the selected diagnostic approach effectively reduces overdiagnosis.

Despite promising feasibility and early diagnostic performance, pilot programs face challenges related to participation rates, resource allocation (especially MRI access), and the need for long-term outcome data to assess mortality benefit and cost-effectiveness. Ongoing European initiatives, such as the PRAISE-U project, are systematically evaluating different pilot models to inform future implementation of organized screening at the population level, with the goal of reducing prostate cancer mortality while minimizing overtreatment and associated morbidity (20).

A recent study has shown the non-inferiority of biparametric MRI compared with multiparametric MRI for the detection of Gleason grade group 2 and higher prostate cancer in biopsy-naïve men (21). This finding provides an opportunity to reduce the potential bottleneck of screening programs by switching to biparametric MRI as it requires fewer resources and allows faster turnover through a screening program.

Overall, the pilot program provided valuable insights into the logistics, challenges, and benefits of organized prostate cancer screening. Our pilot program is still ongoing until we enroll 10 000 men, and we expect that the final analyses will identify the strengths and weaknesses of the health care system. The findings from this initiative will be instrumental in shaping a nationwide screening program that maximizes early detection while minimizing unnecessary interventions. We also expect that a higher number of enrolled men will provide data for cost-effectiveness analyses of similar initiatives for screening prostate cancers.

The CROState pilot program demonstrated a high initial participation rate when GPs led recruitment, suggesting feasibility for broader implementation. The integration of multiparametric MRI and targeted biopsy yielded a high rate of clinically significant cancers while limiting overdiagnosis, with only a small number of cases meeting active surveillance criteria. However, the low rate of repeat PSA testing highlights a key limitation that could undermine diagnostic completeness and program effectiveness.

These findings support the use of GP-led screening pathways and advanced diagnostic tools in prostate cancer screening protocols. Improvements in follow-up adherence and IT integration are necessary before national rollout. While the results are promising, additional data on long-term outcomes, cost-effectiveness, and mortality impact are required before recommending routine implementation in clinical practice.
